# Retracted: Growth Responses and Leaf Antioxidant Metabolism of Grass Pea (*Lathyrus sativus* L.) Genotypes under Salinity Stress

**DOI:** 10.1155/2016/2954587

**Published:** 2016-09-01

**Authors:** International Scholarly Research Notices

International Scholarly Research Notices has retracted the article titled “Growth Responses and Leaf Antioxidant Metabolism of Grass Pea (*Lathyrus sativus* L.) Genotypes under Salinity Stress” [1]. The article was found to contain images with signs of duplication and manipulation in Figures 5(a), 5(b), 6(a), and 6(b), and duplication from Talukdar D. Plant Growth and Leaf Antioxidant Metabolism of Four Elite Grass Pea (*Lathyrus sativus*) Genotypes, Differing in Arsenic Tolerance. Agric Res (2013) 2: 330. doi:10.1007/s40003-013-0085-3 in Figure 6.
